# Effect of bilirubin concentration on the risk of diabetic complications: A meta-analysis of epidemiologic studies

**DOI:** 10.1038/srep41681

**Published:** 2017-01-30

**Authors:** Bo Zhu, Xiaomei Wu, Yifei Bi, Yang Yang

**Affiliations:** 1 Department of Cancer Prevention and Treatment, Cancer Hospital of China Medical University/Liaoning Cancer Hospital & Institute Shenyang, People’s Republic of China; 2 Department of Clinical Epidemiology and Evidence Medicine, The First Hospital of China Medical University, Shenyang, People’s Republic of China; 3 Leiden University Center for Linguistics and Leiden Institute for Brain and Congnition, leiden, zuid-holland, Netherlands; 4 Department of mathematics, School of Fundamental Sciences, China Medical University, Shenyang, People’s Republic of China

## Abstract

Diabetes can affect many parts of the body and is associated with serious complications. Oxidative stress is a major contributor in the pathogenesis of diabetic complications and bilirubin has been shown to have antioxidant effects. The number of studies on the effect of bilirubin on the risk of diabetic complications has increased, but the results are inconsistent. Thus, we performed a meta-analysis to determine the relationship between bilirubin concentration and the risk of diabetic complications, and to investigate if there was a dose-response relationship. We carried out an extensive search in multiple databases. A fixed or random-effects model was used to calculate the pooled estimates. We conducted a dose-response meta-analysis to analyze the association between these estimates. A total of 132,240 subjects from 27 included studies were analyzed in our meta-analysis. A negative nonlinear association between bilirubin concentration and the risk of diabetic complications was identified (OR: 0.77, 95% CI: 0.73–0.81), with a nonlinear association. We also found that there was a negative association between bilirubin concentration and the risk of diabetic nephropathy, diabetic retinopathy and diabetic neuropathy. The results of our meta-analysis indicate that bilirubin may play a protective role in the occurrence of diabetic complications.

Diabetes Mellitus (DM) is an important non-communicable disease, and is a serious threat to human health and global economies. Due to population ageing, urbanization and lifestyle changes, the number of people with DM has increased sharply in both developed and developing countries^1^. The International Diabetes Federation (IDF) reported that the number of individuals with DM was estimated to be 382 million in 2013, and predicted that almost 600 million people would develop this disease by 2035. In the United States, 29.1 million people had DM in 2014^2^, and the United Health Group predicted that approximately half of all Americans would have prediabetes or diabetes by 2020^3^. Approximately 99% of all diabetic patients (28.9 million people aged 20 years or older) have DM. Recently, the burden of DM was shown to be in developing countries rather than developed countries^1^,^4^. The IDF predicted that by 2035, ten countries would have the largest diabetes populations, including four Asian countries–China, India, Indonesia, and Japan—And Asia would be the center of the DM epidemic^4^.

DM has serious effects on many parts of the body, such as the eyes, kidneys, limbs and nervous system. Therefore, diabetic patients can develop many microvascular complications, such as blindness, kidney failure, and lower-limb disorders leading to amputation. From 2005 to 2008, 4.2 million diabetic patients aged 40 years or older developed diabetic retinopathy (DR), which is caused by damage to the small blood vessels in the retina^1^. Diabetic nephropathy (DN) is the leading cause of chronic renal failure and end-stage renal disease^5^. In 2011, 49,677 Americans began treatment for kidney failure due to diabetes^6^. Approximately 73,000 diabetic patients aged 20 years or older underwent non-traumatic lower-limb amputations^6^. Diabetic neuropathy is defined as the presence of symptoms and/or signs of peripheral nerve dysfunctions after exclusion of other causes. It is a heterogeneous condition that encompasses a wide range of peripheral nerve dysfunction and its development may be attributed to diabetes per se or to factors associated with the disease^1^. DM and its complications had become major public health challenge of the 21^st^ century.

As one of the most highly conserved groups of non-polar molecules, bilirubin has been shown to have antioxidant and anti-inflammatory effects^7^,^8^. Bilirubin belongs to a phylogenetically old superfamily of tetrapyrrolic compounds, and is the end product of heme catabolism. Cyclic tetrapyrrole heme is divided into biliverdin, carbon monoxide (CO), and ferrous iron (Fe^2+^) due to the effects of heme oxygenase (HO), which is an enzyme system comprised of two forms: HO-1 and HO-2. Biliverdin is subsequently reduced to bilirubin by biliverdin reductase. Bilirubin concentration increased as higher HO-1 expression increases^9^. An animal study had found that higher HO-1 expression had a protective effect on many diseases^10^. Several studies on the HO-1 pathway have focused on various diseases such as cardiovascular disease, DM and diabetic complications, and evaluated the association between bilirubin concentration and these diseases^11^,^12^,^13^. Initially, the studies on bilirubin were primarily focused on cholestasis, especially in newborns^14^. In recent years, studies on bilirubin found that it played a protective role in vascular diseases. In a prospective study, it was found that the risk of ischemic stroke in the highest bilirubin group was lower than that in the lowest bilirubin group after adjustment for multiple confounding factors (OR: 0.66, 95% confidence interval (CI): 0.49–0.89)^15^. Additionally, Inoguchi *et al*. demonstrated that the prevalence of vascular complications was lower in diabetic patients with Gilbert’s syndrome than that in diabetic patients with normal bilirubin concentration^16^.

Long-term hyperglycemia leads to the generation of reactive oxygen species, mainly by mitochondria. The reaction process occurs in endothelial cells, and led to vascular complications. In diabetic patients, bilirubin may exhibit its potent antioxidant property by inhibiting lipid peroxidation and attenuating low-density lipoprotein (LDL) oxidation^16^. In a new review published in 2016, the authors found that mildly elevated bilirubin concentration had protective effect on an array of diseases (such as cardiovascular disease and diabetes) associated with increased oxidative stress, targeting elevated bilirubin metabolism could be considered a potential therapeutic approach to ameliorate a variety of conditions^17^. The relationship between bilirubin metabolism and occurrence, development and prognosis of disease has become a research focus. In recent years, the number of studies on the relationship between bilirubin concentration and the risk of diabetic complications has increased, some studies have indicated that high bilirubin concentration has protective effect on diabetic complications^18^,^19^,^20^,^21^, but some studies did not find this relationship^22^,^23^. Therefore, it was very necessary to analyze the relationship between bilirubin concentration and the risk of diabetic complications using the meta-analysis method. We also carried out a dose-response meta-analysis to provide a more accurate assessment on the relationship between bilirubin concentration and the risk of diabetic complications.

## Results

### Search results and study characteristics

**Figure 1:**
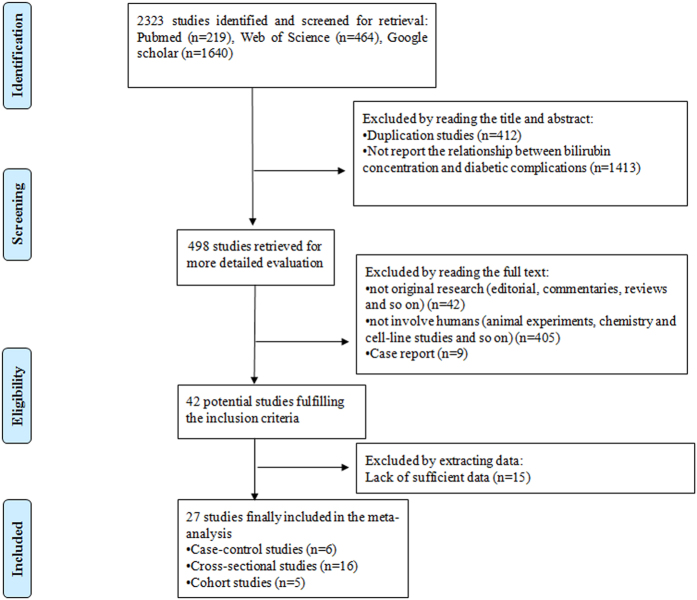
The flow chart of screening progress in our meta-analysis.

Potential studies (2323) were identified from three electronic databases (MEDLINE: 219, Web of Science: 464, and Google scholar: 1640) using search strategy. After reading the title and abstract, we found that 412 studies were duplicates and 1413 studies did not report the relationship between bilirubin concentration and the risk of diabetic complications. Four hundred and ninety-eight studies were read in full, and 471 studies were excluded for various reasons. In total, 27 studies^7^,^18^,^19^,^20^,^21^,^22^,^23^,^24^,^25^,^26^,^27^,^28^,^29^,^30^,^31^,^32^,^33^,^34^,^35^,^36^,^37^,^38^,^39^,^40^,^41^,^42^,^43^ satisfied the inclusion criteria for our meta-analysis. A flow chart of the screening process is shown in Fig. 1.

Table 1 shows the details of the included studies in our meta-analysis. The 27 studies were from five countries (one study was carried out in Australia^7^, thirteen in China^18^,^19^,^24^,^26^,^27^,^29^,^30^,^33^,^34^,^35^,^40^,^43^, one in India^28^, six in Japan^20^,^23^,^25^,^31^,^36^,^42^ and six in Korea^21^,^22^,^37^,^38^,^39^,^41^) and included five cohort studies^7^,^19^,^23^,^31^,^36^, sixteen cross-sectional studies^20^,^21^,^22^,^25^,^30^,^32^,^33^,^34^,^35^,^37^,^38^,^39^,^40^,^41^,^42^,^43^ and six case-control studies^18^,^24^,^26^,^27^,^28^,^29^. Eleven studies assessed DN^19^,^22^,^23^,^24^,^25^,^29^,^31^,^32^,^33^,^40^,^43^, twelve assessed DR^18^,^19^,^25^,^26^,^27^,^28^,^30^,^34^,^35^,^36^,^40^, two assessed diabetic peripheral neuropathy (DPN)^39^,^40^, two assessed diabetic arterial stiffness (DAS)^21^,^43^, one assessed diabetic coronary heart disease (DCH)^43^, one assessed diabetic ischemic stroke (DIS)^43^, one assessed diabetic carotid atherosclerosis (DCA)^42^, one assessed diabetic obstructive coronary artery disease (DOCAD)^38^, one assessed diabetic cardiovascular autonomic neuropathy (DCAN)^37^ and one assessed diabetic amputations (DA)^7^. All the ORs in the included studies were adjusted for the greatest number of potential confounders.

Table 2 shows the basic characteristics of the subjects in the included studies. A total of 132,240 subjects were included in the studies. The study population in each study ranged from 80 to 93,909 and the proportion of males ranged from 32.61% to 100.00%. The mean age of the study population ranged from 48.70 to 87.40 years. The mean BMI of the study population ranged from 21.40 to 26.60 kg/m^2^. The proportion of smokers in the study population ranged from 2.60% to 48.07%, and the proportion of alcohol drinkers in the study population ranged from 7.80% to 51.85%. The mean of duration of DM ranged from 6.14 to 15.19 years.

We assessed 13 biochemical indices which may be related to the occurrence of diabetic complications. Table 3 shows these biochemical indices in the subjects included in the studies.

We assessed the quality of included studies using the quality assessment tool^44^, which was based on a modified Newcastle-Ottawa Quality Assessment Scale (NOS) assessment tool. The quality scores of the included studies are shown in Table 1. The quality scores ranged from seven to eleven and sixteen studies scored eleven points. Eight studies were assessed as moderate quality, and nineteen studies were assessed as high quality.

### Bilirubin and the risk of diabetic complications

**Figure 2:**
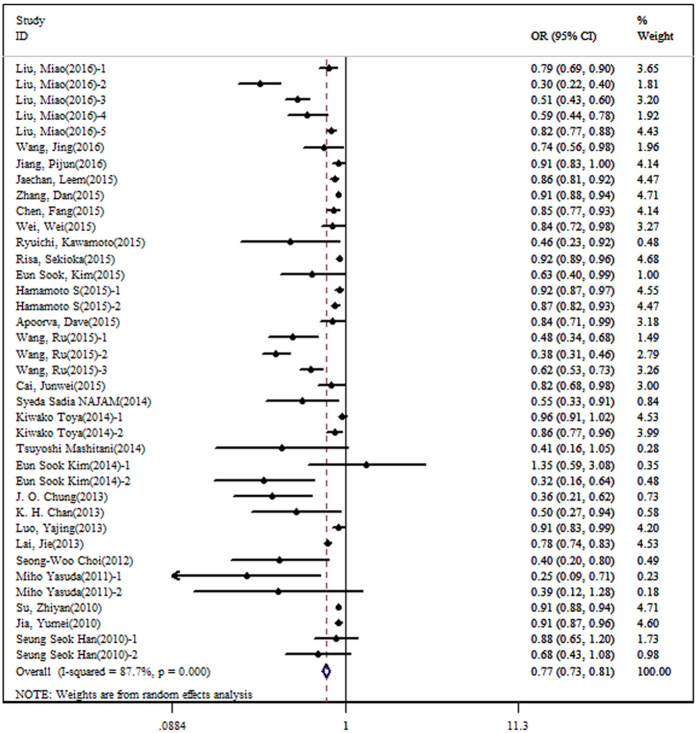
The forest plot on the association between bilirubin concentration and the risk of diabetic complications.

During the process of extracting data, we found that Liu *et al*.^43^ studied several diabetic complications, including DN, DR, DAS, DCH, DIS. Hamamoto *et al*.^25^ studied DR and DN, Wang *et al*.^40^ studied DR, DN and DPN, Kiwako *et al*.^23^ studied microalbuminuria and macroalbuminuria, Eun *et al*.^21^ studied DAS by sex, Miho *et al*.^36^ studied DR in different blood sugar level, and Seung *et al*.^22^ studied DN by sex. Therefore, we extracted 38 sets of statistical data from 27 studies. A negative association between bilirubin concentration and the risk of diabetic complications was observed (OR: 0.77, 95% CI: 0.73–0.81), with high heterogeneity (I^2^ = 87.7%, P < 0.001) (Fig. 2). In order to determine the source of heterogeneity, continuous variables were used in meta-regression, and we found that FBG and HbA1C may be the source of heterogeneity (P value for meta-regression on FBG was = 0.009; P value for meta-regression on HbA1C was = 0.014). Furthermore, we used subgroup analysis with dichotomous variables, and the results also showed that there was a significant association between bilirubin concentration and the risk of diabetic complications. The overall and subgroup results are shown in Table 4.

**Figure 3:**
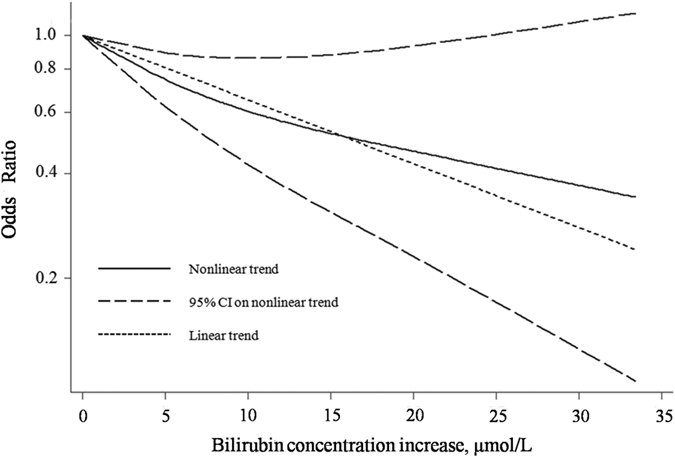
The dose-response association between bilirubin concentration and the risk of diabetic complications.

In the dose-response analysis, we found that 23 sets of statistical data from 13 of the included studies were suitable. A nonlinear association between bilirubin concentration and the risk of diabetic complications was found (P < 0.001). A degree of heterogeneity was observed (P = 0.032). Figure 3 shows the dose–response association between bilirubin concentration and the risk of diabetic complications.

In the sensitivity analysis, we omitted one study at a time, and the pooled OR ranged from 0.76 (95% CI: 0.72–0.80) to 0.79 (95% CI: 0.76–0.83). The results of the sensitivity analysis showed that the results for the effects of bilirubin on diabetic complications were robust.

Both the Begg’s and Egger’s tests showed that publication bias existed in the comparison between bilirubin concentration and the risk of diabetic complications (P value for Begg’s test was <0.001, and P value for Egger’s test was <0.001). Thus, we performed the trim and fill method to identify and correct the asymmetry of the funnel plot, and the results showed a negative association between bilirubin concentration and the risk of diabetic complications (OR: 0.77, 95% CI: 0.73–0.81, P value for heterogeneity was <0.001).

### Bilirubin and the risk of DN

We extracted 13 statistical data sets from 11 studies on DN. We also found that there was a negative association between bilirubin concentration and the risk of DN (OR: 0.79, 95% CI: 0.72–0.87), with high heterogeneity (I^2^ = 88.1%, P < 0.001). We also used subgroup analysis with dichotomous variables and the results show that there was a significant association between bilirubin concentration and the risk of DN. The overall and subgroup results are shown in Table 5. The results of the sensitivity analysis also show that the overall result for the effects of bilirubin on DN were robust, and when we omitted one study at a time, the pooled OR ranged from 0.78 (95% CI: 0.70–0.86) to 0.86 (95% CI: 0.81–0.91).

### Bilirubin and the risk of DR

We extracted 13 statistical data sets from 12 studies on DR. We found that there was a negative association between bilirubin concentration and the risk of DR (OR: 0.84, 95% CI: 0.79–0.89), with moderate heterogeneity (I^2^ = 85.3%, P < 0.001). We also used subgroup analysis with dichotomous variables and the results show that there was a significant association between bilirubin concentration and the risk of DR. The overall and subgroup results are shown in Table 5. The results of the sensitivity analysis also show that the overall result for the effects of bilirubin on DR were robust, and when we omitted one study at a time, the pooled OR ranged from 0.81 (95% CI: 0.76–0.88) to 0.89 (95% CI: 0.86–0.92).

### Bilirubin and the risk of diabetic neuropathy

In this study, diabetic neuropathy included DPN and DCAN. We found that there was a negative association between bilirubin concentration and the risk of diabetic neuropathy (OR: 0.56, 95% CI: 0.43–0.74), with moderate heterogeneity (I^2^ = 43.3%, P = 0.172). As the number of studies on diabetic neuropathy was small, subgroup and sensitivity analyses were not performed.

## Discussion

To the best of our knowledge, this is the first meta-analysis to determine the association between bilirubin concentration and the risk of diabetic complications. We included 27 studies involving 132,240 subjects from five countries. Eleven studies assessed DN, twelve assessed DR, three assessed diabetic neuropathy, and five assessed other complications. The quality of the included studies was relatively high, and more than two-thirds of studies were rated 11 points in our quality assessment. Our results indicated that there was a negative association between bilirubin concentration and the risk of diabetic complications (OR: 0.77, 95% CI: 0.73–0.81), and similar results were also found for DN (OR: 0.79, 95% CI: 0.72–0.87), DR (OR: 0.84, 95% CI: 0.79–0.89) and diabetic neuropathy (OR: 0.56, 95% CI: 0.43–0.74). The dose–response relationship between bilirubin concentration and the risk of diabetic complications was non-liner. Bilirubin showed a protective effect on the risk of diabetic complications.

Bilirubin has potent antioxidant properties, which were first reported in 1954^12^. Due to its antioxidant capacity, bilirubin is oxidized to biliverdin, which is immediately reduced by biliverdin reductase to bilirubin^11^. It suppressed the oxidation of lipids and lipoproteins, especially low-density lipoprotein cholesterol^45^. Compared with the water-soluble antioxidants (such as glutathione), bilirubin has been shown to be more effective at protecting lipids from oxidation^11^. In addition, bilirubin also has anti-inflammatory properties. The *vivo* study on subjects with Gilbert’s syndrome had found that there was a negative association between serum bilirubin concentration and soluble forms of CD40 ligand and P-selectin^46^. Several studies found a negative association between serum bilirubin concentration and C-reactive protein^13^,^47^,^48^. In cardiovascular diseases, several studies found that a negative association between bilirubin concentration and the risk of both coronary and peripheral atherosclerotic disease^49^,^50^,^51^, and a meta-analysis which involved 15000 subjects, demonstrated that increased bilirubin concentration could reduce the risk of cardiovascular diseases^52^.

Diabetes can affect many parts of the body and is associated with serious complications, such as heart disease, stroke, blindness, kidney failure, and lower-limb disorders leading to amputation. Oxidative stress is a major contributor in the pathogenesis of diabetic complications^53^. Kumar *et al*. showed that serum bilirubin concentration was negatively correlated with the level of oxidative stress and positively correlated with the levels of antioxidative enzyme such as superoxide dismutase, catalase, and glutathione peroxidase^54^. Therefore, bilirubin may protect against diabetic complications. However, to date, the mechanism of diabetic complications is still unclear. Possible mechanisms underlying the association between bilirubin and diabetic complications are as follows: high blood sugar can lead to mitochondrial superoxide overproduction in endothelial cells of both large and small vessels, five major pathways, which included polyol pathway flux, increased formation of advanced glycation end products (AGEs), increased expression of the receptor for AGEs and its activating ligands, activation of protein kinase C isoforms, and overactivity of the hexosamine pathway, can be activated by increased superoxide production. These five pathways are involved in the pathogenesis of diabetic complications. Bilirubin not only can inhibit lipid peroxidation and attenuate LDL oxidation, but also reduce the levels of reactive oxygen species. Several studies have analyzed the association between bilirubin concentration and the risk of diabetic complications (such as DN, DR and other diabetic vascular complications)^19^,^38^,^43^,^44^.

For example, in DN, Hamamoto *et al*.^25^ carried out across-sectional study on 523 diabetic patients in Japan. They found that lower bilirubin concentration was an independent risk factor for DN. In DR, Syeda *et al*.^30^ performed a population-based cross-sectional study on 1761 diabetic patients aged ≥40 years in China. The subjects were assigned to quartiles based on serum total bilirubin concentration. The results showed that the prevalence of DR in the entire study population was 9.6%. The prevalence of DR was significantly lower in the highest quartile compared with the other three quartiles. After adjustment for multiple confounding factors, diabetic patients in the highest quartile were less likely to suffer from DR than patients in the lowest quartile. Chung *et al*.^37^ also performed a cross-sectional study in patients with diabetic neuropathy with regard to cardiovascular autonomic neuropathy. The results of this study showed that serum total bilirubin concentration was significantly lower in subjects with cardiovascular autonomic neuropathy. Serum total bilirubin levels were significantly associated with cardiovascular autonomic neuropathy after adjustment for multiple confounders. However, the results on the association between bilirubin concentration and the risk of diabetic complications were inconsistent. Therefore, it was necessary to systematically analyze the relevant studies, and determine their relationship and the key factors that may influence the results of the studies.

In order to ensure that the results of our meta-analysis were both reliable and credible, we carried out the following ways: First, we extracted 38 results on the association between bilirubin concentration and the risk of diabetic complications from the included 27 studies, which helped us to understand the effects of bilirubin on the various complications of DM. The ORs which were extracted from the included studies, were adjusted to take into account the common confounders (such as gender, age and BMI), this ensured the stability of the results of our meta-analysis. Second, the standardized NOS was not suitable for assessing the effects of bilirubin on diabetic complications, therefore, we modified the NOS to satisfy our assessment as the included studies were evaluated more accurately, and this ensured the credibility of the results of our meta-analysis. The included studies did not have a low quality assessment rating, and the results of subgroup analysis were similar to the overall results. Third, we not only analyzed the association between bilirubin concentration and the risk of diabetic complications, but also determined the dose-response relationship, and found that there was a nonlinear association between bilirubin concentration and the risk of diabetic complications. We further analyzed the data for DN and DR. The results were similar to the overall results. This ensured the reliability of the results of our meta-analysis. Fourth, the potential confounders that were extracted contained dichotomous and continuous variables, thus we respectively carried out subgroup analysis and meta-regression, to analyze the effects of the potential confounders on the results of our meta-analysis. We found that FBG and HbA1C may be the source of heterogeneity. Blood glucose control reduced the risk of diabetic complications, especially microvascular (such as eye, kidney and nerve) disease^1^. These results suggested that FBG and HbA1C must be considered confounders which were adjusted in later studies on the association between bilirubin concentration and the risk of diabetic complications.

Although we attempt to ensure that our results were reliable and credible, there were some limitations in our study. First, because the authors of this article did not have access to EMBASE, we could not directly search the relevant articles in this database. To make up for this limitation as much as possible, we searched the relevant articles in Google Scholar. If we found the relevant article in Google Scholar, we purchased the article or sought help online. Second, the source of the subjects in 80% of included studies was hospital-based, these studies may have a certain degree of selection bias as no randomization was performed. We performed subgroup analysis on the source of subjects, and found a negative association between bilirubin concentration and the risk of diabetic complications, and I^2^ in the population-based group was lower than 50%. Third, publication bias was observed, and in order to eliminate this, the trim and fill method was used, and a negative association between bilirubin concentration and the risk of diabetic complications was observed. Fourth, we found that only one study had been carried out in Australia, and the other studies were carried out in Asia. This might lead to selection bias due to race. There was a significant difference in the association between bilirubin concentration and the risk of DA in the Australian study, these results suggested that bilirubin may have an impact on the risk of diabetic complications not only in Asian populations, but also in populations from other counties. We also found that there were only five cohort studies, which accounted for merely 18% of the included studies. Thus, large-sample, long-term cohort studies are needed to validate our results, especially studies from countries outside Asia.

In summary, we collected literature that studied the association between bilirubin concentration and the risk of diabetic complications. Our meta-analysis indicated that higher bilirubin concentration reduced the risk of diabetic complications. This meta-analysis was the first comprehensive quantitative assessment of bilirubin on diabetic complications, suggested that bilirubin may be used as a biomarker of the occurrence of diabetic complications.

## Research Design and Methods

### Search Strategy and Selection Criteria

An extensive search strategy was carried out in multiple databases by October 27, 2016 (MEDLINE, Web of Science, and Google Scholar). In order to collect the relevant articles as many as possible, we set the following key words: bilirubin and (“retinopathy” or “nephropathy” or “neuropathy” or “vascular complication”). We also reviewed reference lists from all the relevant original research and review articles to identify additional possibly eligible studies. There is no language restriction.

### Inclusion and exclusion criteria

According to the inclusion criteria, the possibly relevant articles which had been collected were independently assessed by three reviewers (Xiaomei Wu, Bo Zhu and Yang Yang). Any disagreements were solved by discussion.

The diagnostic criterion for DM, prediabetes and hyperglycemia was used by the World Health Organization (WHO) 1999 criteria^55^ or American Diabetes Association (ADA) 2010 guidelines^56^. On the basis of these diagnostic criterions, DN was defined as urinary albumin excretion ≥30 mg/day (or ≥30 μg/g creatinine), and contained microalbuminuria (urinary albumin excretion between 30 and 300 mg/day or between 30 and 300 μg/g creatinine) and macroalbuminuria (urinary albumin excretion >300 mg/day or >300 μg/g creatinine)^57^. DR was evaluated by an ophthalmologist using fundoscopy exam and contained normal, non-proliferative and proliferative types. Non-proliferative DR showed one or more of the following symptoms: microaneurysm, haemorrhage, exudates, or microvascular abnormalities; proliferative DR showed the generation of new vessels and fibrosis. DPN was defined as patient reporting changes in sensations using the Michigan Neuropathy Screening Instrument (MNSI). DOCAD was defined as ≥50% diameter stenosis in at least one coronary artery. DCAN was assessed by analyzing heart rate response during deep breathing, lying-to-standing and the Valsalva manoeuvre and evaluating blood pressure response to standing^58^. DAS was defined as pulse wave velocity (PWV) ≥1745 cm/s^21^. DA were defined as those above the ankle and minor amputations as those below the ankle^7^. DCH was defined as having been diagnosis one of the following diseases: stable angina, unstable angina, myocardial infarction^43^. DIS was the result of a disturbance of the cerebral circulation which leads ultimately to cell death^43^. DCA was defined as IMT ≥1.0 mm or plaque lesion^42^.

The included studies in our meta-analysis met the following criteria: (1) the study should investigate the relationship between bilirubin concentration and the risk of diabetic complications; (2) the study should contain the odds ratio (OR) with 95% confidence intervals (CIs) on the relationship between bilirubin concentration and the risk of diabetic complications, or had provided available data to calculate the corresponding estimate; (3) if the results of more than two study came from the same population, the latest or highest-quality result was adopted.

The studies were excluded from our meta-analysis if the study did not investigate the relationship between bilirubin concentration and the risk of diabetic complications. The exclusion criteria also contained: (1) the study was not original research, for example editorial, commentary and review; (2) subjects in the study did not involve humans, for example animal experiments, chemistry and cell-line studies; (3) individual case reports; (4) the study did not provide sufficient data to calculate the corresponding estimate on the relationship between bilirubin concentration and the risk of diabetic complications.

### Data extraction and conversion

The included studies were independently read in detail by two reviewers (Bo Zhu and Xiaomei Wu). Two reviewers extracted the information by a table, which was prepared using Microsoft Excel 2007. The information extracted included the following: author, year of publication, country; study design, source of subjects, number of subjects and baseline risk factors (body mass index (BMI), Duration of diabetes, Smoking and alcohol consumption), outcome and its definition, biochemical indexes (fasting blood glucose (FBG), hemoglobin A1c (HbA1C), hypertension, systolic blood pressure (SBP), diastolic blood pressure (DBP), dyslipidaemia, total cholesterol (TC), triglycerides (TG), high density lipoprotein-cholesterol (HDL-C), low density lipoprotein-cholesterol (LDL-C) and serum uric acid (SUA)), the results of statistical analysis. In order to reduce the effects of confounding factors on the pooled estimates, we preferred to extract and analyze the adjusted estimates rather than unadjusted estimates. When the studies only provided categories of bilirubin concentration, the results of the highest category were selected for analysis. For studies, which presented several estimates adjusted for different number of potential confounders, the estimate that adjusted for the most number of potential confounders was selected for analysis.

The units of bilirubin concentration were often used as μmol/L and mg/dL. In order to make unit consistency in all included studies, we converted mg/dL to μmol/L with multiplying by 17.1.

### Quality assessment

The quality of final included studies was independently assessed by two reviewers (Bo Zhu and Xiaomei Wu). There was no standardized quality assessment tool on assessing the effects of bilirubin on diabetic complications, so NOS was modified to satisfy our requirement according to previous study^44^. Six aspects were used to evaluate the quality of the included study, as follows: (1) sample representativeness; (2) sample size; (3) outcome definition and measurement; (4) comparability of results; (5) outcome assessment and (6) statistical methods. The score of study quality ranged from zero to eleven. The study with ten or more points was regard as “high quality”, the study with seven to nine points was regard as “moderate quality”, and otherwise, the study was regarded as “low quality”. The quality assessment tool is shown in Supplementary 1.

### Statistical analysis

In our meta-analysis, we used the pooled odds ratios (ORs) and 95%CIs to analyze the relationship between bilirubin concentration and diabetic complications, which were calculated by using the inverse variance-weighting method. The Chi-square-based Q-test was used to evaluate the heterogeneity among the individual studies. Heterogeneity was quantified based on I^2^, which ranged from 0% to 100% (I^2^ = 0% to 25%, no heterogeneity; I^2^ = 25% to 50%, moderate heterogeneity; I^2^ = 50% to 75%, high heterogeneity; I^2^ = 75% to 100%, extreme heterogeneity). When I^2^ was larger than 50%, a DerSimonian and Laird random-effects model was used; otherwise, the fixed-effects model was used. If there was highly heterogeneity between studies, we used subgroup analysis and meta-regression to find the source of heterogeneity. We performed subgroup analysis by country, number of subjects, design, source of subjects, FBG, HbA1C, the study quality. Restricted maximum likelihood (REML) based random effects meta-regression was carried out to investigate the source of heterogeneity among studies according to the relevant factors. We also used the sensitivity analysis to evaluate the robustness of the results in our meta-analysis. In the sensitivity analysis, we excluded each study in turn and obtained the pooled estimates from the remaining studies. The purpose of sensitivity analysis was to evaluate the effect of a single study on the overall pooled estimates.

We conducted the dose–response meta-analysis to calculate study-specific slopes (i.e., linear trends) and 95%CIs, which proposed by Greenland S. *et al*.^59^ and Orsini N. *et al*.^60^. If the study reported exposure category by a range, the midpoint was calculated by averaging the lower and upper bound; if the lowest category was open ended, the lowest boundary was considered to be zero; if the highest exposure category was open-ended, the width of the open-ended interval was taken to be the same as the adjacent interval.

The possibility of publication bias was assessed using the Begg and Egger’s test. We also performed the Duval and Tweedie nonparametric “trim and fill” procedure to further assess the possible effect of publication bias in our meta-analysis. A two-sided P value < 0.05 in statistical process was considered significantly different. All statistical analysis was performed with the Stata software package (Version 12.0; Stata Corp., College Station, TX).

## Additional Information

**How to cite this article**: Zhu, B. *et al*. Effect of bilirubin concentration on the risk of diabetic complications: A meta-analysis of epidemiologic studies. *Sci. Rep.* **7**, 41681; doi: 10.1038/srep41681 (2017).

**Publisher's note:** Springer Nature remains neutral with regard to jurisdictional claims in published maps and institutional affiliations.

## Supplementary Material

Supplementary Information

## Figures and Tables

**Table 1:** Details of the included studies in our meta-analysis.

Author	Year	Country	Design	Source of subjects	Number of subjects	Outcome	Odds ratio (95% CI)	Adjustment for covariates	Quality of study
Liu, Miao^43^	2016	China	cross-sectional	Hospital-based	1839	DN	0.79 (0.67,0.87)	age, education, marital status, current smoking, current drinking, physical activity ≥0.5 h/day, BMI, hypertension, dyslipidemia, treatment and control status of diabetes	10
						DR	0.30 (0.22,0.40)	age, education, marital status, current smoking, current drinking, physical activity ≥0.5 h/day, BMI, hypertension, dyslipidemia, treatment and control status of diabetes	
						DAS	0.51 (0.41,0.57)	age, education, marital status, current smoking, current drinking, physical activity ≥0.5 h/day, BMI, hypertension, dyslipidemia, treatment and control status of diabetes	
						DCH	0.59 (0.43,0.76)	age, education, marital status, current smoking, current drinking, physical activity ≥0.5 h/day, BMI, hypertension, dyslipidemia, treatment and control status of diabetes	
						DIS	0.82 (0.76,0.87)	age, education, marital status, current smoking, current drinking, physical activity ≥0.5 h/day, BMI, hypertension, dyslipidemia, treatment and control status of diabetes	
Wang, Jing^19^	2016	China	cohort	Population-based	2958	DN	0.74 (0.56,0.98)	age, gender, central obesity, education, smoking status, drinking status, physical activity, SBP, TG, HDL-C, use of medications (hypotensor, lipid-lowering), liver function (ALP, AST and ALT), FBG, use of antidiabetic, duration of diabetes, use of aspirin	11
Ryuichi, Kawamoto^42^	2016	Japan	cross-sectional	Hospital-based	374	DCA	0.46 (0.23,0.93)	age, gender, BMI, smking status, SBP, DBP, Antihepertensive medication, TG, HDL-C, LDL-C, Antidyslipidemic medication, FBG, insulin therapy, eGFR, Uric acid, AST, GGT	9
Jiang, Pijun^24^	2016	China	case-control	Hospital-based	561	DN	0.91 (0.83,1.00)	not list specifically	7
Jaechan, Leem^38^	2015	Korea	cross-sectional	Hospital-based	460	DOCAD	0.86 (0.81,0.92)	age, gender, BMI, duration of diabetes, hypertension, SBP, DBP, current smoking, HbA1C, LDL-C, HDL-C, TG, DR, DN, eGFR, current use of insulin and a statin, AST, ALT and alcohol intake	11
Zhang, Dan^18^	2015	China	case-control	Hospital-based	553	DR	0.91 (0.88,094)	age, gender, duration of diabetes, BMI, HbA1C, LDL-C, TG, SUA and SBP	8
Chen, Fang^26^	2015	China	case-control	Hospital-based	237	DR	0.85 (0.78,0.94)	age, duration of diabetes, FBG, Arteriosclerosis, SBP, UAER	8
Wei, Wei^27^	2015	China	case-control	Hospital-based	100	DR	0.84 (0.72,0.99)	not list specifically	7
Risa, Sekioka^20^	2015	Japan	cross-sectional	Hospital-based	674	DR	0.92 (0.89,0.96)	not list specifically	8
Eun Sook, Kim^39^	2015	Korea	cross-sectional	Hospital-based	1207	DPN	0.63 (0.40,0.99)	age, gender, BMI, duration of diabetes, drinking and smoking status, history of cardiovascular disease, HbA1c, SBP, ALT, hyperlipidemia, eGFR, use of insulin and antihypertensive agents, autonomic neuropathy, DR, and albuminuria	11
Hamamoto S^25^	2015	Japan	cross-sectional	Hospital-based	523	DR	0.92 (0.87,0.97)	age, gender, smoking status	10
						DN	0.87 (0.82,0.93)	age, gender, smoking status	
Apoorva, Dave^28^	2015	India	case-control	Hospital-based	80	DR	0.84 (0.71,0.99)	not list specifically	7
Wang, Ru^40^	2015	China	cross-sectional	Hospital-based	5961	DR	0.48 (0.34,0.68)	age, gender, BMI, duration of diabetes, drinking and smoking status, HbA1C, FBG, ALT, AST, TC, TG and drug treatment	11
						DN	0.38 (0.31,0.46)	age, gender, BMI, duration of diabetes, drinking and smoking status, HbA1C, FBG, ALT, AST, TC, TG and drug treatment	
						DPN	0.62 (0.53,0.73)	age, gender, BMI, duration of diabetes, drinking and smoking status, HbA1C, FBG, ALT, AST, TC, TG and drug treatment	
Cai, Junwei^29^	2015	China	case-control	Hospital-based	102	DN	0.82 (0.69,0.99)	age, BMI, duration of diabetes, SBP, DBP, ALT, FBG, HbA1C, FIN, TG, TC, HDL-C, LDL-C	7
Syeda Sadia NAJAM^30^	2014	China	cross-sectional	Population-based	1761	DR	0.55 (0.33,0.91)	age, gender, current smoking, drinking status, postprandial plasma glucose, HbA1c, DBP, TC, TG, LDL-C and GGT	11
Kiwako Toya^23^	2014	Japan	cohort	Hospital-based	1418	DN (microalbuminuria)	0.96 (0.91,1.02)	age, gender, use of renin–angiotensin–aldosterone system	11
						DN (microalbuminuria)	0.86 (0.77,0.95)	age, gender, use of renin–angiotensin–aldosterone system	
Tsuyoshi Mashitani^31^	2014	Japan	cohort	Hospital-based	957	DN	0.41 (0.16,1.04)	age, gender, BMI, duration of diabetes, follow-up time, SBP, drinking and smoking status, HbA1c, UACR, and use of RAS inhibitors and statins and hemoglobin levels.	11
Eun Sook Kim^21^	2014	Korea	cross-sectional	Hospital-based	1711	DAS	Male:1.35 (0.59,3.07)	age, BMI, duration of diabetes, drinking and smoking status, history of CVD, HbA1c, SBP, DBP, ALT, TC, TG, HDL-C, eGFR, use of insulin, ACEi/ARB, statin, retinopathy and albumin-to-creatinine ratio.	11
						DAS	Female:0.32 (0.16,0.65)	age, BMI, duration of diabetes, drinking and smoking status, history of CVD, HbA1c, SBP, DBP, ALT, TC, TG, HDL-C, eGFR, use of insulin, ACEi/ARB, statin, retinopathy and albumin-to-creatinine ratio.	
J. O. Chung^37^	2013	Korea	cross-sectional	Hospital-based	2291	DCAN	0.36 (0.21,0.63)	age, gender, BMI, smoking habits, AST, ALT, hypertension, hyperlipidaemia, HbA1c, diabetes duration, retinopathy and nephropathy	11
K. H. Chan^7^	2013	Australia	cohort	Hospital-based	9795	DA	0.50 (0.27,0.95)	age, height, smoking status, GGT, HbA1c, and history of previous PAD, non-PAD CVD, amputation or diabetic skin ulcer, neuropathy, nephropathy and diabetic retinopathy, as well as trial treatment allocation	11
Luo, Yajing^32^	2013	China	cross-sectional	Hospital-based	246	DN	091 (0.83,0.99)	duration of diabetes, SBP, HbA1C, FBG, 2hPG, TC, TG	11
Lai, Jie^33^	2013	China	cross-sectional	Hospital-based	435	DN	0.78 (0.74,0.83)	duration of diabetes, SBP, FBG, SUA, Lp (a), hs-CRP, LDL-C, HbA1C	11
Seong-Woo Choi^41^	2012	Korea	cross-sectional	Population-based	690	HbA1c ≥6.5%	0.40 (0.20,0.80)	age, gender, abdominal circumstance, smoking, diabetic duration, hypertension, CCVD history, HDL-C, LDL-C, TG, fasting glucose, eGFR, AST, ALT and GGT.	11
Miho Yasuda^36^	2011	Japan	cohort	Population-based	1672	DR*	0.25 (0.09,0.72)	age, gender, 2hPG, SBP, TC, HDL-C, GGT, history of cardiovascular disease, smoking habits, and alcohol intake.	11
						DR	0.39 (0.12,1.30)	age, gender, 2hPG, SBP, TC, HDL-C, GGT, history of cardiovascular disease, smoking habits and alcohol intake.	
Su, Zhiyan^35^	2010	China	cross-sectional	Hospital-based	664	DR	0.91 (0.88,0.94)	age, gender, duration of diabetes, BMI, WHR, HbA1C, LDL-C, TG, UA, SBP	11
Jia, Yumei^34^	2010	China	cross-sectional	Hospital-based	1062	DR	0.91 (0.87,0.96)	age, gender, duration of diabetes, TC, TG, HDL-C, LDL-C	11
Seung Seok Han^22^	2010	Korea	cross-sectional	Population-based	93909	DN	Male:0.88 (0.64,1.19)	age, BMI, hypertension, TC, TG, HDL- C, and hepatic markers including AST, ALT, alkaline phosphatase, and GGT	11
						DN	Female:0.68 (0.43,1.08)	age, BMI, hypertension, TC, TG, HDL- C, and hepatic markers including AST, ALT, alkaline phosphatase, and GGT	

Note: HbA1C: hemoglobin A1c; SBP: systolic blood pressure; DBP: diastolic blood pressure; TC: total cholesterol; TG: triglycerides; HDL-C: high density lipoprotein-cholesterol, LDL-C: low density lipoprotein-cholesterol; SUA: serum uric acid; eGFR: estimated glomerular filtration rate; ALP: alkaline phosphatase; AST: aspartate aminotransferase; ALT: alanine aminotransferase; GGT: γ-glutamyl transpeptidase; 2hPG: 2-hour post-load plasma glucose; *DR in high blood sugar condition.

**Table 2:** The basic characteristics of the subjects in the included studies.

Study	Male (%)	Age (Year)	BMI (kg/m^2^)	Duration of diabetes (Year)	Smokers (%)	Alcohol drinkers (%)
Liu, Miao (2016)	100	87.40	24.7	n/r	2.60	7.80
Wang, Jing (2016)	45.54	64.07	n/r	n/r	28.74	26.17
Ryuichi, Kawamoto (2016)	45.20	80.00	21.40	n/r	24.60	n/r
Jiang, Pijun (2016)	50.27	60.97	23.56	n/r	n/r	n/r
Jaechan, Leem (2015)	62.17	63.00	25.07	13.34	23.04	n/r
Zhang, Dan (2015)	49.01	59.00	24.81	10.80	n/r	n/r
Chen, Fang (2015)	56.54	58.71	24.89	9.58	35.44	n/r
Wei, Wei (2015)	n/r	59.93	n/r	7.93	0.00	n/r
Risa, Sekioka (2015)	66.17	64.70	25.50	13.90	48.07	n/r
Eun Sook, Kim (2015)	47.80	55.83	25.00	6.93	21.13	38.44
Hamamoto S (2015)	59.66	60.50	24.70	12.20	n/r	n/r
Apoorva, Dave (2015)	n/r	53.93	n/r	7.94	n/r	n/r
Wang, Ru (2015)	60.14	54.13	26.60	9.92	28.38	27.38
Cai, Junwei(2015)	47.06	67.22	25.41	12.48	n/r	n/r
Syeda Sadia NAJAM(2014)	42.87	61.17	26.23	n/r	20.22	10.34
Kiwako Toya(2014)	58.18	59.00	23.70	14.00	n/r	n/r
Kiwako Toya (2014)	60.16	61.00	25.00	15.00	n/r	n/r
Tsuyoshi Mashitani (2014)	63.01	67.10	24.80	14.10	20.38	n/r
Eun Sook Kim (2014)-Male	n/r	55.20	24.60	7.00	38.04	56.63
Eun Sook Kim (2014)-Female	n/r	58.80	25.10	8.10	5.09	17.59
J. O. Chung (2013)	64.99	59.01	24.38	9.24	17.55	32.52
K. H. Chan (2013)	61.72	62.00	n/r	n/r	9.41	n/r
Luo, Yajing (2013)	47.97	62.05	26.60	10.76	28.86	n/r
Lai, Jie (2013)	54.02	63.10	25.29	15.19	n/r	n/r
Seong-Woo Choi(2012)	32.61	68.20	24.50	8.90	15.36	n/r
Miho Yasuda (2011)*	53.35	64.17	23.93	6.14	20.87	51.85
Miho Yasuda (2011)	n/r	n/r	n/r	n/r	n/r	n/r
Su, Zhiyan (2010)	49.25	59.70	24.90	9.17	32.83	n/r
Jia, Yumei (2010)	53.77	58.16	n/r	7.33	n/r	n/r
Seung Seok Han (2010)-Male	n/r	n/r	n/r	n/r	n/r	n/r
Seung Seok Han (2010)-Female	n/r	n/r	n/r	n/r	n/r	n/r

^*^DR in high blood sugar condition; n/r: not reported.

**Table 3:** The biochemical indicators of the subjects in the included studies.

Study	FBG (mmol/L)	HbA1C (%)	Hypertension (%)	SBP (mmHg)	DBP (mmHg)	Dyslipidaemia (%)	TC (mmol/L)	TG (mmol/L)	HDL-C (mmol/L)	LDL-C (mmol/L)	SUA (μmol/L)
Liu, Miao (2016)	7.0	n/r	67.7	133.3	72.6	38.7	4.90	1.80	1.90	2.50	n/r
Wang, Jing (2016)	8.23	n/r	45.30	131.3	77.27	n/r	5.27	n/r	1.37	3.10	n/r
Ryuichi, Kawamoto (2016)	n/r	n/r	9.10	136.00	74.00	n/r	n/r	0.90	1.42	2.75	n/r
Jiang, Pijun (2016)	10.41	9.33	n/r	134.78	74.17	n/r	4.69	2.03	1.17	2.70	307.72
Jaechan, Leem (2015)	n/r	n/r	54.13	132.91	78.00	n/r	4.53	1.37	1.27	2.83	n/r
Zhang, Dan (2015)	n/r	8.81	n/r	135.32	113.07	n/r	n/r	1.64	1.22	3.22	n/r
Chen, Fang (2015)	9.78	9.98	50.63	129.95	77.87	n/r	4.64	2.36	1.06	n/r	n/r
Wei, Wei (2015)	n/r	n/r	n/r	n/r	n/r	n/r	n/r	n/r	n/r	n/r	n/r
Risa, Sekioka (2015)	n/r	9.13	73.89	n/r	n/r	77.15	n/r	n/r	n/r	n/r	n/r
Eun Sook, Kim (2015)	n/r	8.44	n/r	129.90	78.39	31.07	n/r	2.01	1.19	2.65	n/r
Hamamoto S (2015)	n/r	9.60	n/r	132.69	n/r	n/r	n/r	n/r	n/r	n/r	n/r
Apoorva, Dave (2015)	n/r	n/r	n/r	n/r	n/r	n/r	n/r	n/r	n/r	n/r	n/r
Wang, Ru (2015)	8.18	8.45	n/r	136.22	79.68	n/r	4.65	1.98	1.10	2.88	n/r
Cai, Junwei (2015)	9.61	7.86	n/r	125.55	76.06	n/r	6.11	2.05	1.05	2.71	n/r
Syeda Sadia NAJAM (2014)	7.01	6.50	45.32	149.11	84.16	n/r	5.50	1.68	1.27	3.29	n/r
Kiwako Toya (2014)	n/r	8.00	n/r	132.00	76.00	n/r	n/r	n/r	1.42	n/r	n/r
Kiwako Toya (2014)	n/r	8.30	n/r	139.00	76.00	n/r	n/r	n/r	1.32	n/r	n/r
Tsuyoshi Mashitani (2014)	n/r	7.60	n/r	134.20	74.50	n/r	5.00	1.70	1.50	2.90	321.30
Eun Sook Kim (2014)-Male	n/r	8.20	n/r	129.00	79.70	n/r	4.59	2.64	1.14	2.50	n/r
Eun Sook Kim (2014)-Female	n/r	n/r	n/r	n/r	n/r	n/r	n/r	n/r	n/r	n/r	n/r
J. O. Chung (2013)	8.38	8.52	68.66	127.22	77.23	64.82	4.68	1.80	1.20	2.80	n/r
K. H. Chan (2013)	n/r	n/r	56.58	n/r	n/r	n/r	n/r	n/r	n/r	n/r	n/r
Luo, Yajing (2013)	9.87	8.93	n/r	149.53	81.57	n/r	5.13	2.03	n/r	3.05	289.55
Lai, Jie (2013)	9.31	8.10	n/r	136.27	n/r	n/r	4.76	1.85	1.22	3.00	328.39
Seong-Woo Choi (2012)	7.60	7.40	64.64	130.30	72.00	n/r	5.01	2.06	1.23	2.85	n/r
Miho Yasuda (2011)*	6.21	5.37	58.49	135.29	81.96	n/r	5.48	n/r	1.70	n/r	n/r
Miho Yasuda (2011)	n/r	n/r	n/r	n/r	n/r	n/r	n/r	n/r	n/r	n/r	n/r
Su, Zhiyan (2010)	n/r	9.22	n/r	134.70	80.00	n/r	4.95	1.66	1.20	3.19	308.77
Jia, Yumei (2010)	9.31	9.38	n/r	n/r	n/r	n/r	4.98	2.13	1.51	2.70	n/r
Seung Seok Han (2010)-Male	n/r	n/r	n/r	n/r	n/r	n/r	n/r	n/r	n/r	n/r	n/r
Seung Seok Han (2010)-Female	n/r	n/r	n/r	n/r	n/r	n/r	n/r	n/r	n/r	n/r	n/r

^*^DR in high blood sugar condition; n/r: not reported.

**Table 4:** The pooled ORs on the association between bilirubin concentration and the risk of diabetic complications.

	No. of study data	Model for meta-analysis	OR (95%CI)	I^2^ (%)	P for heterogeneity
Overall	38	R	0.77 (0.73, 0.81)	87.7	<0.001
**Subgroup Analysis**					
**Country**					
China	19	R	0.73 (0.68, 0.79)	92.4	<0.001
Korea	8	R	0.65 (0.49, 0.84)	72.0	0.001
Japan	9	R	0.90 (0.85, 0.95)	62.1	0.007
Else	2	R	0.71 (0.44, 1.14)	59.1	0.118
**Number**					
<2000	30	R	0.82 (0.79, 0.86)	84.4	<0.001
≥2000	8	R	0.57 (0.45, 0.71)	78.4	<0.001
**Design**					
Case-control	5	F	0.90 (0.87, 0.93)	0.0	0.453
Cross-sectional	26	R	0.73 (0.68, 0.78)	90.7	<0.001
Cohort	7	R	0.78 (0.65, 0.93)	70.8	0.002
**Source of subjects**					
Hospital-based	31	R	0.78 (0.74, 0.82)	89.4	<0.001
Population-based	7	F	0.63 (0.49, 0.81)	41.7	0.113
**FBG**					
<9.00	13	R	0.53 (0.43, 0.64)	90.5	<0.001
≥9.00	6	R	0.86 (0.81, 0.92)	74.3	0.002
**HbA1C**					
<9.00	16	R	0.70 (0.63, 0.79)	90.6	<0.001
≥9.00	7	F	0.91 (0.89, 0.93)	0.0	0.622
**NOS**					
Moderate quality	8	F	0.90 (0.87, 0.93)	21.8	0.257
High quality	30	R	0.72 (0.67, 0.77)	89.7	<0.001

R, random-effects model, F: fix-effects model.

**Table 5:** The pooled ORs on the association between bilirubin concentration and risk of DN and DR.

	The pooled ORs on the association between bilirubin concentration and risk of DN					The pooled ORs on the association between bilirubin concentration and risk of DR				
	No. of study data	Model for meta-analysis	OR (95%CI)	I^2^ (%)	P for heterogeneity	No. of study data	Model for meta-analysis	OR (95%CI)	I^2^ (%)	P for heterogeneity
Overall	13	R	0.79 (0.72, 0.87)	88.1	<0.001	13	R	0.84 (0.79, 0.89)	85.3	<0.001
**Subgroup Analysis**										
**Country**										
China	7	R	0.75 (0.64, 0.87)	91.6	<0.001	8	R	0.80 (0.73, 0.87)	90.2	<0.001
Korea	2	F	0.81 (0.63, 1.05)	0.0	0.363	0	—	—	—	—
Japan	4	R	0.89 (0.82, 0.97)	67.5	0.026	4	R	0.89 (0.81, 0.93)	62.6	0.046
**Number**										
<2000	9	R	0.86 (0.81, 0.92)	75.2	<0.001	12	R	0.86 (0.81, 0.91)	84.0	<0.001
≥2000	4	R	0.63 (0.41, 0.99)	89.1	<0.001	1	—	0.48 (0.34, 0.68)	—	—
**Design**										
Case-control	2	F	0.89 (0.82, 0.97)	1.0	0.315	3	F	0.89 (0.85, 0.94)	22.8	0.274
Cross-sectional	7	R	0.74 (0.64, 0.86)	91.5	<0.001	8	R	0.81 (0.75, 0.89)	90.1	<0.001
Cohort	4	R	0.87 (0.76, 0.99)	66.0	0.032	2	F	0.30 (0.14, 0.66)	0.0	0.581
**Source of subjects**										
Hospital-based	10	R	0.80 (0.72, 0.88)	90.9	<0.001	10	R	0.85 (0.81, 0.91)	87.1	<0.001
Population-based	3	F	0.78 (0.64, 0.94)	0.0	0.589	3	F	0.46 (0.30, 0.71)	0.0	0.392
**FBG**										
<9.00	3	R	0.61 (0.37, 0.99)	94.7	<0.001	4	F	0.40 (0.28, 0.56)	55.8	0.079
≥9.00	4	R	0.85 (0.78, 0.94)	75.8	0.006	2	F	0.89 (0.84, 0.95)	37.7	0.205
**HbA1C**										
<9.00	7	R	0.76 (0.65, 0.89)	93.7	<0.001	4	R	0.56 (0.34, 0.93)	86.7	<0.001
≥9.00	2	F	0.88 (0.84, 0.93)	0.0	0.433	5	F	0.91 (0.89, 0.93)	0.0	0.643
**NOS**										
Moderate quality	2	F	0.89 (0.82, 0.97)	1.0	0.315	5	F	0.91 (0.88, 0.93)	3.7	0.386
High quality	11	R	0.78 (0.70, 0.87)	89.8	<0.001	8	R	0.72 (0.63, 0.82)	90.9	<0.001

R, random-effects model, F: fix-effects model.
