# Supplementary material for: Effect of bilirubin concentration on the risk of diabetic complications: A meta-analysis of epidemiologic studies
Source: Sci Rep. 2017 Jan 30;7:41681. doi: 10.1038/srep41681 (PMC5278382; doi:10.1038/srep41681)
Supplement: Supplementary Information [file srep41681-s1.pdf]

Effect of bilirubin concentration on the risk of diabetic complications: A meta-analysis of epidemiologic studies

Bo Zhu[1,*], Xiaomei Wu[2], Yifei Bi[3], Yang Yang[4]

[1] Department of Cancer Prevention and Treatment, Cancer Hospital of China Medical University/ Liaoning Cancer Hospital & Institute Shenyang, People's Republic of China

[2] Department of Clinical Epidemiology and Evidence Medicine, The First Hospital of China Medical University, Shenyang, People's Republic of China

[3] Leiden University Center for Linguistics and Leiden Institute for Brain and Congnition, leiden, zuid-holland, Netherlands

[4] Department of mathematics, School of Fundamental Sciences, China Medical University, Shenyang, People's Republic of China

[*]Corresponding author

E-mail: 15998896991@163.com

Supplementary 1: Modified Newcastle-Ottawa Quality Assessment tool

1. Sample representativeness (maximum 3 points)
   a. truly representative (3 points)
   b. somewhat representative (2 points)
   c. random sample of participants (1 point)
   d. no description

2. Sample size (maximum 1 point)
   a. sample size was sufficiently large and justified (1 point)
   b. not satisfactory

3. Outcome definition and measurement (maximum 2 points)
   a. clear case and control group definition were provided and a validated method was used to do this (2 points)
   b. a non-validated method was used but was suitable with sufficient justification (1 point)
   c. no description

4. Comparability of results (maximum 2 points)
   a. study attempts to control for important risk factors (2 points)
   b. study controls for other factors/other exploration of results (1 point)
   c. no attempt was made to further explore results

5. Outcome assessment (maximum 2 points)
   a. independent association established (2 points)
   b. unadjusted association (1 point)
   c. no description

6. Statistical tests (maximum 1 point)
   a. suitable (1 point)
   b. not suitable

Total maximum 11 points
